# A Historical Review of Indian Perspectives on Techniques of Tympanoplasty

**DOI:** 10.1155/2020/1408270

**Published:** 2020-03-30

**Authors:** Manu Malhotra, Rashmi Malhotra, Saurabh Varshney, Madhu Priya, Abhishek Bhardwaj, Amit Tyagi, Amit Kumar, Shubhankur Gupta

**Affiliations:** ^1^Department of Otolaryngology, All India Institute of Medical Sciences, Veerbhadra Marg, Rishikesh, Uttarakhand, PIN 249201, India; ^2^Department of Anatomy, All India Institute of Medical Sciences, Veerbhadra Marg, Rishikesh, Uttarakhand, PIN 249201, India

## Abstract

Reconstructive surgery of the conductive hearing mechanism is collectively called as tympanoplasty, which has gradually evolved over time with the contributions from all over the word. The aim of the present historical review is to summarize the Indian contributions in the development of the technique of tympanoplasty. The literature review was conducted using only the “*Medline*” search using keywords “tympanoplasty” and “ossiculoplasty” in “India” on 15^th^ June 2016. A total of 195 articles and abstracts were found dated from the year 1998 onwards. Articles describing work on technique were included, and those describing only experimentation with graft material were excluded. All articles were fully read and analysed. It was found that there had been experiments regarding the choice of anaesthesia and the use of combinations of different chemical agents for this purpose. There were suggestions in favour of monitored anaesthesia care for the surgery in select patients. Surgeons expressed their perspectives on the time and conditions for the surgery, laterality of surgery, different types of incisions, use of endoscopes, graft placement techniques, ossicular replacements with autologous or allogenic grafts, and the timing of prophylactic antibiotic therapy given after or during the surgery. The range of work is wide and covers most of the aspects of surgery; however, the incorporation of a uniform methodology and standards reporting results were lacking in the articles reviewed.

## 1. Introduction

The surgery of tympanoplasty, which involves repair of middle ear hearing apparatus, has evolved from the basic techniques of repair of the eardrum, which we call as myringoplasty. Banzer was the first to attempt repair of the perforated tympanic membrane in 1640. He used a pig's bladder stretched across an ivory tube and placed it in the ear [1]. Toynbee in 1853 placed a rubber disc attached to a silver wire and kept over the perforation resulting in hearing improvement [1]. Sir William Wilde (1853) published a procedure for postaural incision and mastoid cortex removal [1]. Blake in 1877 placed a paper patch on a tympanic membrane perforation and observed hearing improvement in many patients [1]. The first true tympanoplasty was performed by Berthold in 1878 using the de-epithelised tympanic membrane [2]. The new era of modern tympanoplasty began with the advent of operating the microscope, microscopic instruments, and antibiotics in the 1950s. Zöllner and Wullstein had earlier described overlay techniques using skin grafts [3, 4]. In the 1960s, the overlay technique consisted of removing the surface epithelium of the tympanic membrane and placement of the graft lateral to the perforation [1]. Shea first originally described underlay technique using the vein and the fascia. Several authors reported underlay technique scores over the overlay technique [5, 6]. Various techniques besides the above such as sandwich, crown cork, swing door, laser assisted, microclip, fascial pegging, annular wedge, loop, and umbrella graft tympanoplasty have been described as subsequent modifications of the above [7–20].

The first ossicular reconstruction was done by Hall and Ryztner in 1957 with autograft ossicles [21, 22]. The use of alloplastic material for reconstruction of the middle ear mechanism can be traced back to 1952 when Wullestein used an oval strut of vinyl acrylic or “palavit” as an acoustic transmitter between the footplate of stapes and the tympanic membrane [23]. In the late 1950s and the 1960s, biocompatible material, such as polyethylene tubing, Teflon, and Proplast, was used. In the late 1970s, a high-density polyethylene sponge was developed. Wehrs in 1972 used homograft ossicles for reconstruction of the ossicular chain. Later in 1989, Yung designed hydroxyapatite prosthesis in order to reduce preparation time [24]. It has been reported that more than 54 alloplastic materials have been marketed for ossicular reconstructions [24, 25]. Titanium, which was introduced by the American otolaryngologists in late the 1990s, was actually used in a significant number of patients in Germans in 1993. For the last decade, titanium has become a popular material in ossicular implants [25].

There had been several experiments in India with the techniques of tympanoplasty, though no historical review has been written so far specifically highlighting these. The aim of the present historical review is to summarize the Indian contributions in the development of the technique of tympanoplasty. The literature review was conducted using only “*Medline*” search using keywords of “tympanoplasty” and “ossiculoplasty” with “India,” respectively, on 15^th^ June 2016. A total of 195 articles and abstracts were found dated from the year 1998 onwards. On further analysis, only 52 were found relevant for the review article. The content of each abstract was studied in order to identify studies relevant to each topic. All chosen articles were fully read, and their references were also examined for articles of relevance. Since Medline articles were available from 1998 onwards, articles published before that were not included. It was found that, during the study, there had been several experiments with the graft material used for tympanoplasty, and there is substantial literature on the subject which deserves a separate review. It was, therefore, decided by the authors not to consider it in the current article. Rather than providing a chronological account of development of tympanoplasty in India, the author would like to present a summarized review under the headings: (1) Anaesthesia for tympanoplasty, (2) Technique of Surgery, (3) Laterality of Surgery, (4) Antibiotic Coverage, and (3) Conclusions.

### 1.1. Anaesthesia for Tympanoplasty

Otology is an important surgical specialty where the quality of anaesthesia can influence the field of interest and its outcomes. Tympanoplasty has been performed under the effect of monitored local anaesthesia or with general anaesthesia; however, some otologic surgeons prefer to give infiltration of 2% lidocaine with 1 : 200,000 adrenaline for infiltration even when the patient is under general anaesthesia [26]. Senthil et al. in 2012 in a prospective comparative study reported that infiltration of 2% lidocaine with 1 : 200,000 adrenaline has a significant effect over the grade of bleeding in the operative field and also on first one-hour postoperative pain relief. They used Boezaart's grading system for recording preoperative bleeding and visual analogue scale for postoperative pain in patients having chronic otitis media, who were randomly allocated in two groups [27].

Monitored anaesthesia care (MAC) has been defined by American Society of Anesthesiologists as a specific anaesthesia service, meant for a diagnostic or therapeutic procedure done under local anaesthesia along with sedation and analgesia [28]. Commonly used medications for monitored local anaesthesia were benzodiazepines, opioids, and propofol [28]. Parikh et al. in 2013 published the reports of a prospective double-blind study comparing dexmedetomidine and combination of midazolam-fentanyl for tympanoplasty in ninety patients under monitored local anaesthesia and concluded that dexmedetomidine is comparable to midazolam-fentanyl combination for sedative effects and analgesia in tympanoplasty with better surgeon and patient satisfaction [29]. Another comparative study results were reported by Sen and Sen in 2014, who in a comparative study, proved that fortwin-Phenergan-midazolam combination is superior to the ketamine-midazolam combination. This study was however conducted on patients undergoing different surgeries such as septoplasty, lip repair, dacrocystectomy, and cataract surgery, besides tympanoplasty, whose number of cases were only eighteen out of fifty [28]. Thota et al. in 2015 published a report in which forty patients undergoing tympanoplasty randomly received either propofol or midazolam with fentanyl and local anaesthesia with 2% lignocaine and 1 : 200,000 adrenaline. They concluded that propofol shows faster recovery and less nausea vomiting than the midazolam group though both agents were found to be safe, simple, versatile, and provide excellent sedation and with rapid trouble-free recovery [31]. Most articles justified the use of MAC for tympanoplasty as an effective alternative of general anaesthesia in a suitable consenting patient.

### 1.2. Techniques of Surgery

In 1998, Pusalkar et al. described the use of a cross-slit gold bell which is connected with a double-band titanium cuff by a tiny gold wire. The prosthesis was crimped on the stamp of the long process with open titanium bands; the bell was kept over the head of stapes. The authors reportedly implanted 81 such prosthesis and observed that the preoperative hearing loss which averaged between 30 and 40 dB improved to average of 15 dB hearing loss in 40 patients and 10 dB in 23 patients [32].

Roychaudhuri in 2004 performed tympanoplasty in 450 patients having subtotal and large perforations with anterior bony overhang or a very small rim of perforation. After removing the margin of the tympanic membrane remnant, three flaps (superior, anterior, and posterior) were elevated from the external auditory canal wall. Temporalis fascia was then placed over the handle of the malleus, and all three flaps were repositioned over it. The authors had claimed 94% success rates in terms of graft take-up rates, and air-bone gap closure was 10 dB in 87% cases and 25 dB in 13% cases [33].

Desarda et al. in 2005 performed reconstructive tympanoplasty in 600 cases of safe and unsafe chronic otitis media. They divided subjects into four study groups depending upon the extent and type of disease and surgery required. In the first group (*n* = 300) of myringoplasty done with on-lay technique, the graft take up was 96%. In the second group (*n* = 110) where different levels of ossiculoplasty were performed using the tragal cartilage shaped in “L,” “T,” “bow,” and “boomerang” shape the graft take-up rate was reportedly 84%. Audiometric results showed 15–20 dB of air-bone gap closure. The third group (*n* = 70) was called the ossiculoplasty group as several osseous defects such as attic, posterosuperior quadrants, posterior canal wall, and annular defects were closed by tragal perichondrium and cartilage grafts. In this group, the authors reported 75% success, while 25% patients had to undergo revision surgery. In the fourth and last group, mastoid obliteration was done with tragal perichondrium and cartilage-covered pedicled temporalis muscle. Here, the success rates were still lower to 70% [34].

Mishra et al. conducted a prospective study on 100 cases having subtotal perforations. They performed type I tympanoplasty in all cases through the postaural route, using circumferential incision in the external auditory canal extending from 12'o clock to 1'o clock, just medial to the spine of Henle, to raise a superiorly based tympanomeatal flap. They achieved 97% graft take-up rates and 95% closure of air-bone gap to 10–30 dB [35].

Mahadevaiah in 2008 described a technique of modified intact canal wall mastoidectomy in which they thinned out the anterior aspect of the posterior canal wall including the posterior bony rim by drilling and removing the outer attic wall also. Thereafter, reconstruction of the tympanic membrane was done with the temporalis fascia and attic with composite graft. Autologous or allogenic ossicles with septal cartilage were used for ossiculoplasty. The authors argued that since the posterior canal wall was thinned from inside, sinus tympani could be approached easily, and opening of facial recess was not required. This technique was tried in 126 patients, out of which 69.1% achieved serviceable hearing (air-bone gap < 20 dB) [36].

Vijayendra et al. advocated that canalplasty, in order to widen the external auditory canal by drilling till the bony annulus becomes visible, should be considered an integral part of tympanoplasty. In year 2008, they published their work in which they compared the hearing gain of tympanoplasties conducted without canalpasty to those done with canalplasties and concluded that we can achieve an additional hearing gain of 9 dB with the latter [37].

Nagle et al. in 2009 compared the results of type I tympanoplasty in 50 cases with dry mucosal disease and 50 cases of mucosal disease with scanty mucoid discharge. In all the patients, they performed type I tympanoplasty by underlay technique. In dry ears, they recorded the graft take-up rate of 88%, while in wet ears, the graft success rate was 74%, and the difference however they wrote was statistically insignificant (*p* > 0.5). The difference in hearing improvement ranging from 0 to 30 dB of air-bone gap was also insignificant between the two groups [38].

Breaking through the traditional mould of doing tympanoplasty under microscopic vision, Yadav et al. insisted on using endoscopes for visual assistance during the surgery. They reasoned that endoscopes provide all round vision of all hidden areas in the middle ear, and it may not be necessary to raise the tympanomeatal flap while doing tympanoplasty. In 2009, they reported a study of 50 cases where only the cases of safe chronic otitis media with small- and medium-sized perforation were included. The results showed graft take-up rate of 80% and an air-bone gap closure of 10 dB in 80% of the cases which had healed successfully [39]. Potentiating the idea of endoscope-assisted tympanoplasty, Mohindra and Panda published their work in which they had carried out 17 grommet insertions, 49 myringoplasties, and 6 ossiculoplasties with the help of endoscope. They reported 91% graft take-up and more than 20 dB average air-bone gap closure in their patients [40].

Malhotra et al. in a series of three articles, one published in the year 2010 and two in 2014 tried to turn tables on titanium total ossicular replacement prosthesis (TORP) with an autologous prosthesis which they called “Umbrella Graft.” They initially described a design in which the vault of prosthesis was constructed from the conchal cartilage, and the stalk was drilled out from the autologous malleus. The assembly was constructed in such a fashion that no tissue glue was required for its stability and the stalk would rest on the footplate of stapes, while the vault would rest against the grafted neotympanic membrane. The assembly was stabilized at two points of contact, i.e., footplate and tympanic membrane. Cartilage vault with its perichondrium gave strength to the new eardrum, and the stalk conveyed the sound to the footplate. It was reported that, out of 22 patients, 77.3% benefitted in terms of reduction of the air-bone gap by at least 10 dB or more. The authors however improved their design in their subsequent publication and preserved the malleus by using the drilled-out cortical bone in its place, as stalk. This study included 40 patients and displayed better hearing results than with the previous version. It was reported that an overall mean improvement in the air-bone gap was 24 dB approximately, and successful rehabilitation of pure tone average to 30 dB or less was achieved in 75% of patients, and air-bone gap to 20 dB or less was attained in 82.5% of patients. In the other study, they also described the use of a total ossicular replacement prosthesis constructed from a block of the cortical bone, by drilling it into a shape of an umbrella. Here, the successful rehabilitation of the air-bone gap to 20 dB or less was achieved in 95% of patients, and an overall mean improvement in the air-bone gap was 26 dB approximately. The data were presented as per the guidelines of the American Academy of Otolaryngology-Head and Neck Surgery Committee on Hearing and Equilibrium [18, 41, 42].

Kasbekar et al. in 2015 reported a study in which they performed cartilage augmentation in cases with Charachon stage II and III retractions of the tympanic membrane. During surgery, the disease in the retraction pocket or the middle ear was cleared and if spreading into the attic atticotomy was performed. The facial recess was exposed, and the sinus tympani was inspected for disease with endoscope. Separate thinned-out cartilage pieces were placed in different areas of retraction. They finally suggested that retractions of the tympanic membrane if treated surgically with cartilage augmentation can reduce the advancement of disease and give acceptable hearing results [43].

Sohil Vadiya in 2015 published a study on 84 patients which underwent type I tympanoplasty. In all these, patients were having a dry central perforation, and the manubrium of malleus was medially rotated, touching the medial wall of the middle ear. The patients were divided into two groups. In view of providing advantage of lever ratio by slightly freeing the handle of malleus and hence correcting the foreshortening, the author cut the tendon of tensor tympani, in the first group. In the second group, no such procedure was done, and the tendon of tensor tympani was not released. After postoperative evaluation, it was concluded that, though the graft take-up rates in both of the groups were the same, the hearing improvements were found to be significantly better in the group where correction of foreshortening was done. No other study from the Indian author under mentioned criteria of search was found to have published on this topic [44].

### 1.3. Laterality of Surgery

Most surgeons do not routinely perform bilateral tympanoplasty in the same sitting. This is primarily because of the risk of iatrogenic sensorineural loss. The evidence in favour of these restrictive arguments was as scarce as the evidence against it till Mane et al. published their reports in 2013. They conducted bilateral type I tympanoplasties in 14 patients (28) ears and achieved graft take-up rates as high as 96% with the air-bone gap closure up to 20 dB in all the ears [45]. In the same year, Raghuwanshi and Asati reported 93% graft take-up rates in 32 patients (64 ears) [46]. Rai et al. in 2014 refined their method in validating the concept of bilateral tympanoplasty by creating a control group and an experimental group, both having 60 patients each, undergoing type I tympanoplasty. They obtained no significant statistical difference between the two groups and concluded that bilateral tympanoplasty is as safe as unilateral tympanoplasty. It is noteworthy that, in all three studies, no complication of sensorineural hearing loss was found [47].

### 1.4. Antibiotic Usage

The duration and timing of giving antimicrobials to patients undergoing tympanomastoid surgeries had long been the topic of debate. Bidkar et al. in 2014 conducted a comparative study in which they administered parenteral perioperative antimicrobials to the first group of 39 patients undergoing tympanoplasty with cortical mastoidectomy and extended oral antimicrobials postoperatively for 8 days to the second group of 39 patients who had undergone the same surgery. Outcomes evaluated on the basis of wound infection and graft take-up rate revealed that there was no additional benefit in giving extended antimicrobial prophylaxis in these patients [48].

## 2. Conclusion

The results of all the studies that have been reviewed as per our search protocols have either suggested some modifications in the techniques or have tried a new technique which have been summarized in Table 1. It is evident that the surgeons have experimented with almost all the aspects of surgery including time and conditions for surgery, laterality of surgery, different types of incisions, use of endoscopes, graft placement techniques, ossicular replacements with autologous or allogenic grafts, and the timing of prophylactic antibiotic therapy given after or during the surgery, and the outcome of reports shows that the results in terms of graft take-up rates and hearing improvement are comparable to those published in rest of the world literature; however, the incorporation of a uniform methodology and standards in reporting results are lacking. It is the recommendation of the authors that common guidelines for reporting results of tympanoplasty be framed for the Indian subcontinent as a whole. Such an effort will promote uniform reporting standards and hence will become a stable step stone for future analysis and research in the middle ear reconstruction.

## Figures and Tables

**Table 1:** Techniques and results.

No.	Author	Year	Technique innovation/recommendations	Graft take-up rates (%)	ABG^*∗*^ of 10–30 dB (%)
1.	Pusalkar et al.	1998	Cross-slit gold bell with titanium cuff over the head of stapes	NR^*∗∗*^	77
2.	Roychaudhry	2004	Three strip tympanomeatal flaps	95	100
3.	Desarda et al.	2005	Tragal cartilage shaped in “L,” “T,” “bow,” and “boomerang” shape for ossiculoplasty	70–96	70–96
4.	Mishra et al.	2007	12'o clock to 1'o clock tympanomeatal flap	97	95
5.	Mahadevaiah	2008	Modified intact canal wall-up mastoidectomy	91.3	69.1
6.	Vijayendra	2008	Compulsory canalplasty with tympanoplasty	100	NR^*∗∗*^
7.	Nagle et al.	2009	Wet ear tympanoplasty	74	100
8.	Yadav et al.	2009	Endoscopy-assisted tympanplasty	80	80
9.	Mohindra and Panda	2009	Endoscopy-assisted tympanplasty	91	91
10.	Mane	2013	Bilateral tympanoplasty	96	96
11.	Raghuvanshi and Asati	2013	Bilateral tympanoplasty	93	91.6
12.	Raj et al	2014	Bilateral tympanoplasty	90–93	90–94
13.	Malhotra et al.	2010, 2014	Umbrella graft/autologous TORP	95–100	77–97.5
14.	Kasbekar et al.	2015	Cartilage augmentation with modified tympanoplasty	NR^*∗∗*^	NR^*∗∗*^
15.	Vadiya	2015	Sectioning of the tensor tympani muscle in type 1 tympanoplasty	95.24	93

^*∗*^ABG = air-bone gap; ^*∗∗*^NR = not reported in this format.
